# Chromosome arm-specific BAC end sequences permit comparative analysis of homoeologous chromosomes and genomes of polyploid wheat

**DOI:** 10.1186/1471-2229-12-64

**Published:** 2012-05-04

**Authors:** Sunish K Sehgal, Wanlong Li, Pablo D Rabinowicz, Agnes Chan, Hana Šimková, Jaroslav Doležel, Bikram S Gill

**Affiliations:** 1 Wheat Genetic and Genomic Resources Center, Department of Plant Pathology, Kansas State University, Manhattan, KS 66506, USA; 2 Department of Biology and Microbiology, South Dakota State University, Brookings, SD 57007, USA; 3 Institute for Genome Sciences, University of Maryland School of Medicine, Baltimore, MD 21201, USA; 4 The J. Craig Venter Institute, Rockville, MD 20850, USA; 5 Institute of Experimental Botany, Centre of the Region Haná for Biotechnological and Agricultural Research, Sokolovska 6, Olomouc CZ-77200, Czech Republic; 6 Faculty of Science, Genomics and Biotechnology Section, Department of Biological Sciences, King Abdulaziz University, Jeddah 21589, Saudi Arabia

## Abstract

****Background**:**

Bread wheat, one of the world’s staple food crops, has the largest, highly repetitive and polyploid genome among the cereal crops. The wheat genome holds the key to crop genetic improvement against challenges such as climate change, environmental degradation, and water scarcity. To unravel the complex wheat genome, the International Wheat Genome Sequencing Consortium (IWGSC) is pursuing a chromosome- and chromosome arm-based approach to physical mapping and sequencing. Here we report on the use of a BAC library made from flow-sorted telosomic chromosome 3A short arm (t3AS) for marker development and analysis of sequence composition and comparative evolution of homoeologous genomes of hexaploid wheat.

****Results**:**

The end-sequencing of 9,984 random BACs from a chromosome arm 3AS-specific library (TaaCsp3AShA) generated 11,014,359 bp of high quality sequence from 17,591 BAC-ends with an average length of 626 bp. The sequence represents 3.2% of t3AS with an average DNA sequence read every 19 kb. Overall, 79% of the sequence consisted of repetitive elements, 1.38% as coding regions (estimated 2,850 genes) and another 19% of unknown origin. Comparative sequence analysis suggested that 70-77% of the genes present in both 3A and 3B were syntenic with model species. Among the transposable elements, gypsy/sabrina (12.4%) was the most abundant repeat and was significantly more frequent in 3A compared to homoeologous chromosome 3B. Twenty novel repetitive sequences were also identified using *de novo* repeat identification. BESs were screened to identify simple sequence repeats (SSR) and transposable element junctions. A total of 1,057 SSRs were identified with a density of one per 10.4 kb, and 7,928 junctions between transposable elements (TE) and other sequences were identified with a density of one per 1.39 kb. With the objective of enhancing the marker density of chromosome 3AS, oligonucleotide primers were successfully designed from 758 SSRs and 695 Insertion Site Based Polymorphisms (ISBPs). Of the 96 ISBP primer pairs tested, 28 (29%) were 3A-specific and compared to 17 (18%) for 96 SSRs.

****Conclusion**:**

This work reports on the use of wheat chromosome arm 3AS-specific BAC library for the targeted generation of sequence data from a particular region of the huge genome of wheat. A large quantity of sequences were generated from the A genome of hexaploid wheat for comparative genome analysis with homoeologous B and D genomes and other model grass genomes. Hundreds of molecular markers were developed from the 3AS arm-specific sequences; these and other sequences will be useful in gene discovery and physical mapping.

## **Background**

Plant genomes may vary over 2,000-fold in genome size (*Genlisea aurea,* ~64 Mb to *Paris japonica,* ~149 Gb). Within the grass family, bread wheat, also known as common or hexaploid wheat (*Triticum aestivum* L., 2n = 6x = 42), has a large genome size of 17 Gb because of two allopolypoidization events and also a very high repetitive DNA content (reviewed in Gill et al. [1]). Each chromosome of wheat, at an average size of 809 Mb (17 Gb/21), is twice the size of the rice genome (390 Mb). Therefore, the physical mapping and sequencing of the wheat genome poses a technical challenge and is cost prohibitive, as discussed at an NSF-sponsored workshop [1]. The wheat genomics community decided on a chromosome-based approach to physical mapping and sequencing to overcome these problems and to provide an opportunity for division of labor and cost sharing by establishing an international consortium of scientists (http://www.wheatgenome.org/). This approach is feasible because of the ability to purify wheat chromosomes and/or chromosome arms by flow cytometric sorting. Particular chromosomes and chromosome arms can be isolated at over 90 % purity and in sufficient quantity for making chromosome and chromosome arm-specific BAC libraries [2-4].

Among the wheat chromosomes, 3B is the largest, sorts out as a single peak on a flow karyotype and its BAC library [5] was used to construct the first wheat chromosome physical map [6]. Another peak contains three of the smallest wheat chromosomes 1D, 4D and 6D, and their physical map construction is underway (http://wggrc.plantpath.ksu.edu/wheat/Dgenome/). For the remaining wheat chromosomes, lines carrying telocentric chromosomes, which represent individual arms, must be used to increase sorting resolution and library construction [2,3]. Chromosome and chromosome arm-specific BAC libraries now have been constructed for most of the wheat chromosomes (http://lmcc.ieb.cz/dna-libraries/cereals)[4].

Flow-sorted chromosomes and libraries have been utilized for generating chromosome and chromosome arm-specific sequence information for gene discovery and comparative genome analysis. BAC end sequences (BESs) have been analyzed from BAC libraries and can be highly informative in determining genome content and organization, enhance the value of BACs as a genomic resource and also provide random sequence information. BESs have been used to estimate the distribution of the repetitive elements including the retrotransposons, DNA transposons and SSRs in several plant species [7-14]. Paux et al. [9] generated 11 Mb of random BESs from chromosome 3B of bread wheat and reported 86% of the sequences as repetitive elements, 1.2% as coding region and 13% was unknown. BESs can be an excellent source for marker development for genetic and physical mapping. BESs were used to develop simple sequence repeats, genic sequence-based markers and, more recently, inserted transposable elements junction based ISBP markers [9,10,13-15].

Sequences from the whole-genome amplification of flow-sorted chromosomes or chromosome-arms have been published for several wheat and barley chromosomes [16-22]. Wicker et al. [19] sequenced flow-sorted wheat group-1 chromosomes by Roche/454 technology at 1.3–2.2x coverage and used the sequence data to estimate gene syntenic relationships of the Triticeae with *Brachypodium*, rice and sorghum and suggested a large number of sequences that are nonsyntenic to model grasses are probably pseudogenes. Berkman et al. [17,21] recently sequenced the flow sorted chromosomes 7BS and 7DS of wheat to isolate the low copy and genic sequences and observed ~ 60% rate of colinearity with *Brachypodium*. Recently, Rustenholnz et al. studied the gene space organization in wheat by mapping the expressed portion of chromosome 3B using barley microarrays [23] and a wheat unigene microarray [24] and observed a 2-fold increase in gene density from centromere towards the telomeres. A BES [9] and transcript map [24] of chromosome 3B provide an opportunity for comparative analysis of homoeologous chromosomes.

In this study, we used the first arm-specific library in wheat from chromosome 3AS [25] for which we are developing a physical map. The 3AS BAC library consists of 55, 296 clones (physical map 3ASv1.0; http://wggrc.plantpath.ksu.edu/wheat/3A/3A_index.html). We randomly selected nearly 10,000 BACs and end sequenced them to generate 11 Mb of sequence. These sequences were analyzed to obtain insight into the sequence composition of wheat chromosome arm 3AS and its evolutionary relationship with other homoeologous chromosomes and model grass genomes. Furthermore, we identified DNA motifs potentially useful as molecular markers to saturate chromosome 3AS.

## **Results and discussion**

### **BAC-end sequencing and data processing**

Chromosome 3A of bread wheat is among the largest wheat chromosomes with a metaphase size of 11.8 μm, is submetacentric with an arm ratio of 1.3 and accounts for 14.4% of the total A genome of hexaploid wheat [26]. Chromosome arm 3AS is estimated at ~355 Mb, equivalent to 0.8 times the size of the rice genome. We have fingerprinted and assembled contigs of 47,063 clones of the bread wheat cultivar Chinese Spring chromosome arm 3AS-specific BAC library (physical map 3ASv1.0; http://wggrc.plantpath.ksu.edu/wheat/3A/3A_index.html). The first phase of assembly of the 3AS physical map used FPC 8.5.2 [27], where 35,124 clones are assembled into 1,677 contigs with the remaining 11,939 clones existing as singletons. In parallel, a total of 9,984 random BACs were end-sequenced and, after preliminary screening for length and quality assessments, a total of 18,022 high-quality BESs were generated with success rate of over 90%. Among them, 431 (2.3%) sequences were eliminated after masking vector sequences and sequences contaminated with bacterial and organelle genomes using CROSS_ MATCH and BLASTN searches [28,29]. In total, 17,591 BESs were generated with an average size of 626 bp. The total BESs length was 11,014,359 bp with a GC content of 44.5%, which suggests that wheat is AT rich, similar to other cereal genomes [10,30,31]. The BESs constituted 3.2% of chromosome arm 3AS. The distribution of forward and reverse BESs displayed a 1:1 ratio (9045 F and 8997R). Tracing back the 9,984 end-sequenced BAC clones to the first phase assembly of the 3AS fingerprint map revealed that 6,506 clones are located in 1,071 contigs and 3,688 singletons (Table 1). On an average, six BACs were end-sequenced per contig, theoretically providing coverage of one BES per 45.4 kb across the chromosome arm. All BESs generated herein have been deposited in the GenBank databases under the “GenBank Accession” EI: EI666997-EI676076 and ER772249-ER781190.

**Table 1:** Origin and distribution of 3AS BAC clones used in BAC end sequencing in the first FPC assembly of chromosome 3AS of bread wheat

**BACs**	**No. of end-sequenced BACs**	**Average number of end sequenced BACs/Contig**
Contigs	6,506	6
Size >200 kb	1,926	7
Size >500 kb	3,447	19
Singletons	3,478	1
Total	9,984	-

### **Analysis of the repeat fraction and identification of novel repeat elements**

The 17,591 BESs accounting for 11 Mb of t3AS were analyzed sequentially for their repeat and gene content using semi-automated pipeline. Based on similarity searches against a repeat database, 79.1% of the nucleotide sequence corresponded to repetitive sequences (Table 2). The class I TEs (retroelements) constitute 66.9% of the sequence, followed by 4.1% for class II TEs (DNA transposons). Within the class I TEs, long terminal repeat (LTR) retrotransposons were the most prominent elements accounting for 98.8%.The most common repeat families of class I were *gypsy*-like (12,150 reads), followed by *copia*-like LTR retrotransposons (3,368 reads). Non-LTR retrotransposons, such as LINE (222 reads, 0.7%) and SINE (1 read), were observed at a much lower frequency (Table 2). Clearly, LTR retrotransposons overshadow non-LTR retrotransposons both in number of matches in BESs and percent of sequence. The next most abundant repeats were the class II TEs DNA transposons with 1,626 BESs showing homology. The CACTA family was the most abundant type of DNA transposon accounting for 84.6 % of the class II elements.

**Table 2:** Occurrence and distribution of repetitive DNA in the BAC end sequences of bread wheat chromosome arm 3AS

**Class, subclass, family**	**No. of hits**	**No. of bases (bp)**	**Fraction of chromosome arm 3AS (%)**
Class I elements (retrotransposons)	15,913	7,370,015	66.9
LTR retrotransposons	15,665	7,284,593	66.1
*gypsy*-like	12,150	5,614,688	50.9
*copia*-like	3,368	1,615,408	14.6
TRIM	16	3,931	0.0
Unknown	131	50,566	0.5
Non-LTR retrotransposons	248	85,422	0.8
LINE	222	77,153	0.7
SINE	1	75	0.0
Unknown	25	8,194	0.1
Class II elements (DNA transposons)	1,626	450,920	4.1
CACTA	1,220	381,611	3.5
Mutator	112	24,277	0.2
MITE	264	35,028	0.3
LITE/Pong	20	7,055	0.1
Unclassified elements	10	2,949	0.0
Other known repeats	311	15,832	0.1
Simple repeats	291	13,983	0.1
Tandem repeats	20	1,849	0.0
Low complexity	219	10,947	0.1
Unknown repeats	332	829,211	7.5
Total	18,558	8,712,367	79.1

**Table 3:** The frequency and distribution of SSRs in wheat homoeologous chromosomes The SSRs on chromosome arm 3AS were obtained from BESs analysis and SSRs from chromosome 3B similar analysis from BESs [9]

	**Wheat 3AS (355 Mb)**	**Wheat 3B (1000 Mb)**
Dinucleotides	779 (73.8%)	324 (18.2%)
Trinucleotides	268 (25.2%)	943 (52.9%)
Tetranucleotides	10 (1.0%)	515 (28.9%)
SSR frequency	10.4 kb	6.1 kb

Public repeat databases were used to mask the BESs matching to known DNA repeat sequences. However, the repeat detection could be limited by the size and diversity of the repeat database, such as the absence of genome and species-specific elements. Furthermore, the rapid evolution of repetitive DNA elements may lead to the origin of several unknown repetitive DNA elements. To identify unknown and wheat-specific repetitive DNA elements, the BESs were masked with known repetitive elements and then compared to each other. The BESs with multiple hits (more than eight) at high stringency (at least 100 bp aligned contiguously) were identified as repeat sequences. Families of repeat sequences were obtained computationally from BESs and then subjected to multiple sequence alignment to obtain 122 consensus sequences. The consensus sequences were merged at overlapping regions and extended using CAP3 software [32] and detected 31 putative novel repeats with sizes ranging from 199 bp to 1,420 bp (Additional file 1). The putative repeat elements could be considerably longer but, because the average BES length was 623 bp, only a part of the repeats could be identified. The searching of all databases, including TREP, RepBase and Genbank, against putative repeats resulted in the identification of 11 protein coding genes representing large gene families, 10 of which also matched transposable proteins. The remaining 20 repeat elements showed no similarity in BLASTN or BLASTX [33] and were termed as *Triticum* novel repeat sequences (TRES). The novel, repeat sequences (TRES) were analyzed for their presence in wheat chromosome 3A by another BLAST against BESs. These TRES were repeated at a higher frequency compared to the initial search in the BESs because of the extended length of the repeat sequences. TRES2 had maximum number of hits in the BESs (84 at e <10^-25^), and the novel repeats totaled 84 matches in the BESs database (Additional file 2). Seventeen of the 20 TRESs had a hit on chromosome 3B BESs when BLASTN searched in NCBI nt/nr database.

### **Gene content and functional annotation**

After masking the known and putatively new repetitive sequences, the remaining BESs were used to estimate the gene content of chromosome arm 3AS. The sequences were compared with wheat transcript assemblies (*Triticum aestivum* release 2) using TBLASTX and BLASTN algorithms [33]. We found 519 unique sequences that were similar (e-value 10^-50^) to an EST with a cumulative match length of 152,662 bp, representing about 1.38 % of the data set. Considering the size of 3AS as 355 Mb, the estimated transcribed fraction of 1.38 % corresponds to 4.8 Mb in 3AS and 11.4 Mb for the entire 3A chromosome. Analysis of full-length cDNAs in several grass species have shown the average lengths of FLcDNA varying between 1,000-2,000 bp. In 37,139 *Oryza sativa* and 7,455 maize FLcDNAs, the average length was found to be 1,746 ± 945 bp [34] and 1,442 bp [35], respectively. In the Triticeae tribe, the average length of 22,651 Uni-FLcDNAs in barley was 1,711 ± 863 bp [34]. Considering 1.7 kb as the average length of a gene based on the average in barley, our results suggest the presence of about 2,850 genes on the short arm of chromosome 3A and ~6,700 genes on chromosome 3A. This estimate is a little lower than the prediction of 7–8,000 genes in chromosome 3A based on preliminary results from Roche/454 sequences of sorted 3A chromosome arms (our unpublished results).

The gene estimates for the homoeologous 3B chromosome, which is 20% bigger than 3A, varies from 6,000 genes based on BES analysis [9] to over 8,400 to 9,500 genes, based on sequence analysis of 17 Mb of contiguous BAC sequences [36] and ~6,300 genes based on 1.2x coverage of sorted chromosome 3B by Solexa/Illumina sequencing [36]. The estimated number of genes on chromosomes 3A and 3B is much higher than the 4,856 genes predicted for syntenic rice chromosome 1 [30]. However, the predicted gene numbers may be overestimated, because it is difficult to distinguish *bona fide* genes from pseudogenes or gene fragments due to the incompleteness of genome or chromosome sequence data.

**Figure 1:**
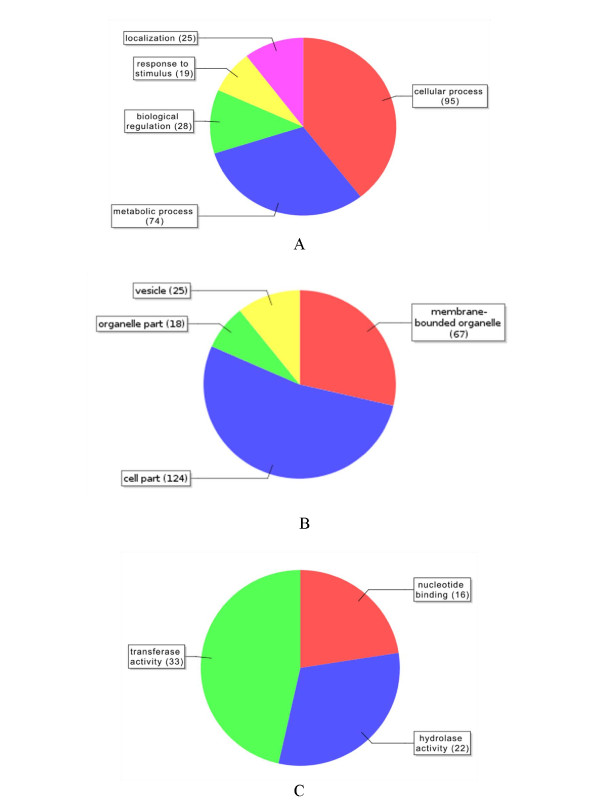
** Distribution of annotated sequences for the chromosome 3A BAC ends under the gene ontology classifications carried out with the automated software Blast2GO.** GO-annotated blast hits (1,928) were assigned into the ontology classifications biological processes, cellular components and molecular functions. (A) Biological process. A total of 241 sequences were identified. Categories expressed as a percentage of the total are cellular process (39.4%), metabolic process (30.7%), biological regulation (11.6%), response to stimulus (7.8%) and localization (10.3%). (B) Cellular component. A total of 234 sequences were identified. Categories expressed as a percentage of the total are membrane-bounded organelle (28.6%), cell part (52.9%), organelle part (7.6%) and vesicle (10.6%). (C) Molecular function. A total of 71 sequences were identified. Categories expressed as a percentage of the total are nucleotide binding (22.5%), hydrolase activity (30.9%) and transferase activity (46.4%). The GO classification is based on the biological process class of an associate GO term with an alpha score of at least level 0.6 and ontology level 2 of Blast2GO [37].

Repeat-masked BESs were functionally annotated with BLASTX searches against the NCBI non-redundant database and functionally classified using Blast2GO [37]. A total of 1,928 BESs had a hit in the database, of which 1,477 sequences (76%) had a definite hit, 109 sequences (5%) hit a transposon and another 342 sequence (18%) hit a hypothetical or predicted protein (Additional file 3). Of 1,928 sequences, only 864 were assigned to GO categories, and an enzyme code was identified for only 31 sequences (Additional file 3). Chromosome 3AS BESs were classified into three categories: biological processes, molecular functions and cellular components. A total of 241 sequences were classified as biological processes, 234 were cellular components and 71 were of metabolic function (Figure 1). Within the biological process classification, the largest categories were cellular processes (39.3% of the sequences) and metabolic processes (30.7% of the sequences), followed by biological regulation (11.6% of the sequences) (Figure 1A). For the cellular components (Figure 1B), 52.8% of the sequences were cell parts and 28.6% were membrane-bounded organelles. Within the molecular functions class (Figure 1C), 46%, 30%, and 22% of the sequences were categorized as transferase activity, hydrolase activity and nucleotide binding, respectively.

### **Comparative sequence composition of homoeologous A, B and D genomes**

The homoeologous genomes of wheat have undergone dramatic sequence diversification in the intergenic regions, but have still retained the backbone of gene synteny with their ancestral genomes. A significant amount of representative sequence data was generated from chromosome 3A in the present study and from chromosome 3B [9,24] and the D genome [38]. Assuming that a chromosome arm could act as a good representative sample of the genome, we utilized 11 Mb BESs each from chromosome arm 3AS, chromosome 3B [9] and a 2.9 Mb of shotgun sequence from the D genome [38] to compare the composition of homoeologous genomes. Our estimate of the repeat content in chromosome 3AS (79.1%) is similar to the 73-82% repeat content of other A-genome chromosomes sequenced by next generation technology from flow-sorted chromosome-arm specific MDA DNA[19,20,22]. However, MDA is known to introduce a bias in the representation of individual amplified regions [39,40], and a more accurate estimation must await quantitative analysis of the repeat content from flow-sorted chromosome arms DNA without the MDA step. However, BES information from chromosomes 3AS and 3B are the best available sequence samples to compare with homoeologous chromosomes and genomes and, together with next generation sequencing of wheat chromosomes 1A, 4A, 5A, 1B, 7BS, 1D and 7DS, provide a snapshot of the gene content of wheat.

**Figure 2:**
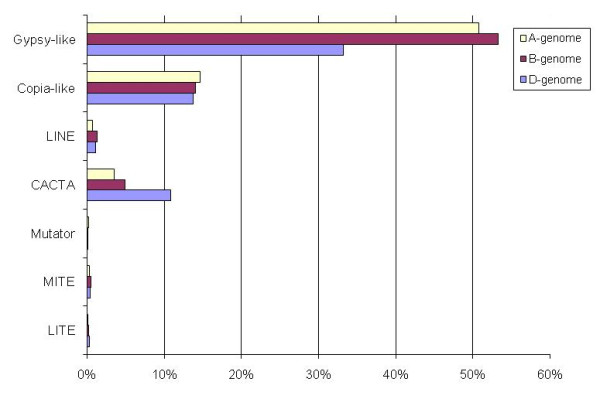
** Histogram showing the comparative abundance of different retrotransposons and DNA transposon families (data expressed in per cent of genome) in the three homoeologous genomes of wheat.** The BAC end sequences for chromosome arm 3AS (A genome) and comparison with the B and D genomes. The data representative for the B and D genomes was obtained in a similar way, using 10.8 Mb of BESs from wheat chromosome 3B [9], and the D genome was represented by 2.9 Mb sequence obtained from whole-genome, shotgun sequence form *Ae. tauschii* the D-genome donor of bread wheat [38].

The total repeat fraction in the A genome was 79.1%, which was less than that of the B genome (85.9%) and higher than that of the D genome (74%), indicating a positive correlation between TE content and genome size. The most abundant retrotransposons in all three genomes were *gypsy*-like, accounting for 33-53% in the A, B and D genomes, followed by *copia*-like (Figure 2). The A genome has a higher percentage of *copia*-like elements compared to the B and D genomes, however, the B genome is richest in *gypsy*-like elements. Interestingly, DNA transposons, such as CACTA elements, are in significantly higher proportion in the D genome compared to the A and B genomes (Figure 2). The Sabrina and Fatima elements seem to be the most abundant and have played a key role in expansion of the A genome along with other elements, such as Erika, Angela and Wilma, which form a considerable part of the retroelements in the genome (Additional file 4). For chromosome 5A, Sabrina (18%), Wilma (8.2%) and Fatima (6.3%) were the most abundant repeats [20]. The Sabrina, Angela, Barbara,Vagabond, Egug and Erika elements were significantly higher (p-value < 0.0001) in the A genome compared to the B genome based on BESs analysis. The ratio of class I elements to class II elements in the A (16.2) and B (12.3) genomes is quite similar, whereas it is significantly lower (4.1) in the D genome, indicating the significant role of DNA transposons in D-genome expansion.

Based on the analysis of 3B BESs and the reanalysis of the D-genome shotgun sequence [38], Paux et al.[9] hypothesized that the Triticeae genomes have evolved from an ancestral genome of approximately 1 Gb. The portion of the genome not related to the repetitive sequences in the A genome based on 3AS and 5A sequence data is estimated at 1.1 Gb (~20% of the 5.5 Gb A genome). However, most probably, this is an overestimate, because the wheat repeated sequence database is incomplete and the non-repetitive portion of the D genome based on evidence from bioinformatics and hybridization analyses was just 8.4% [38].

GC content is an important feature and was quite comparable between chromosomes 3AS (44.5%) and the other A-genome chromosomes 1AL (44.7%, [14]) 4AS (44.7%, [22]) however, the GC content of chromosome 4AL (41.4%, [22]) was considerably lower possibly because of biasness caused by MDA. The GC content in the B-genome chromosome 3B (44.6%, [9]) was quite similar to that of the A-genome chromosomes of wheat, and the GC content of wheat is comparable to other grasses, such as rye (45.9%, [10]), maize (46.5%, [31]) and rice (44.0%, [30]).

The BESs from chromosome 3AS were BLASTN against the 3,000-loci transcript map recently developed for chromosome 3B [24], and 100 BESs matched to 77 unigenes from chromosome 3B. Of these, 77 BESs (matching 77 unigenes), 45 had a hit in rice (40/77) or *Brachypodium* (40/77) with 70% (28/40) of the hits in rice and 77% (31/40) in *Brachypodium* in syntenic regions (Additional file 5). Rustenholz et al. [24] reported 35-42% of unigenes genes mapped on chromosome 3B were non-syntenic in model grasses genomes. Our results, based on the sequence from 3A together with 3B unigenes [24] showed that 23-30% of the genes are non-syntenic in wheat, which is consistent with data from chromosome arms 1AL, 1BL, and 1DL that showed 20-45% were non-syntenic [19]. A similar rate of colinearity was observed for chromosomes 7BS and 7DS with the corresponding region of the *Brachypodium* by sequence analysis of sorted chromosome arms [21]. This erosion of colinearity could be explained by a high rate of transposable element activity in hexaploid wheat, such as TE-driven gene movement [19]. Nearly, 40% (32/77) BESs having a hit to 3B unigenes had no hit in rice or *Brachypodium*. Rustenholz et al. [24] reported that 89% of these unigenes were expressed, and we found 11 of the 32 BESs had a hit with barley full-length cDNA suggesting these genes are less likely to be pseudogenes. Most probably, these genes are less conserved and, therefore, remain undetected in model genomes, however, a few of them could be novel wheat/Triticeae-specific genes. In the recent barley genome sequence analysis by Meyer et al. [18], similar results were observed where 23% of the barley genes had no sequence similarity in model genomes, although they were expressed in barley.

Based on our BES analysis, we estimated one gene per 123 kb and ~44,600 genes in the A genome of wheat. Vitulo et al. [20] recently sequenced the MDA from flow-sorted chromosome 5A of wheat and estimated 5,088 genes of chromosome 5A and with a gene density of one gene per 162 kb. The coding fraction for chromosome 3AS (1.38%) was comparable to those of chromosome 5A (1.08-1.30%, [20]) and 1AL (1.03%, [14]). In another sequence analysis and comparison between the chromosome 4A of wheat and model genome *Brachypodium*, Hernandez et al. [22] estimated 9,500 genes on chromosome 4A and ~61,500 in the A genome. However, taking into account the large number of pseudogenes and paralogs, they estimated a minimum number of 4,300 genes on chromosome 4A and 28,000 in the A genome of wheat [22]. The number of genes estimated in our analysis is higher than conservative estimates based on chromosomes 4A and 5A, however, it corresponds well with the recently estimated numbers of genes in the A genome based on BESs from chromosome 1AL (50,000 genes [14]) and the number of genes in the B genome based on 17 Mb of BAC sequence of chromosome 3B (41,000-50,000 genes [36]). These estimates are much lower than predicted in earlier studies based on the random sequencing of methylfiltrated libraries [41] and significantly higher than the estimated 36,000 genes for the D-genome donor *Ae. tauschii* based on BAC sequencing [42].

Berkman et al. [21] made a very stringent estimate of about 25,000-26,000 genes per genome of wheat based on sequence analysis of wheat chromosome arms 7BS and 7DS. However, the estimates made by Berkman et al. [21] and Hernandez et al. [22] are based on the genome zipper approach. These estimates do not take into account about 23-30% of the genes that lack sufficient sequence similarity to any gene in the three model grass genomes as suggested by Meyer et al. [18] in barley and our analysis of 3AS BESs and 3B unigenes in wheat.

On the other hand, the estimate of gene content by several other studies show that the relatively higher gene content in wheat (100,000-350,000) [9,41] could be an inflation in the actual number of genes due to misannotations of pseudogenes as all these estimates are based on partial gene sequences or a relatively small data set compared to the large wheat genome. Furthermore, recent sequence analysis of the group-1 chromosomes of wheat showed that a large number of nonsysntenic genes are present in only one of three wheat genomes and only 60 % of them showed transcriptional evidence, suggesting that most of them are pseudogenes [19] making annotation of genes based on sequence similarity suboptimal. We may have a better estimate of the gene content once full-length gene sequences and a gold standard reference sequence of wheat becomes available allowing us to differentiate between genes and gene fragments.

### **Comparison to model grass genomes**

**Figure 3:**
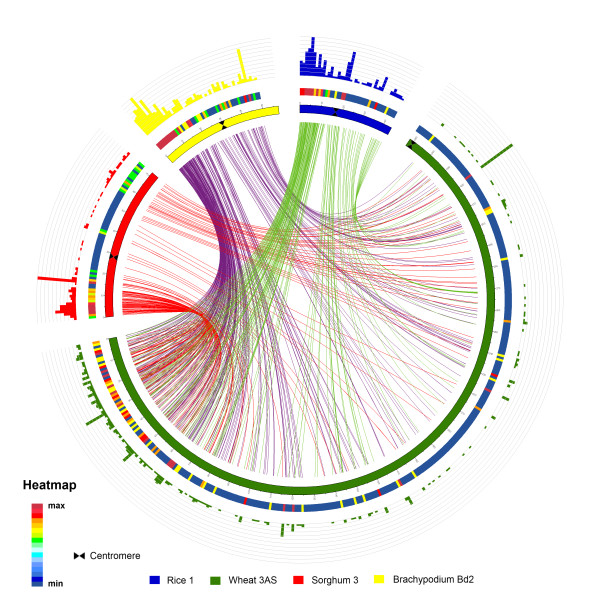
** High-resolution comparative analysis between wheat chromosome 3AS and rice chromosome 1,*Brachypodium*Bd2 and*Sorghum*chromosome 3.** High-density comparative analysis chromosome 3AS BAC end sequences versus the sequenced model grass genomes of rice, *Brachypodium* and *Sorghum*. The figure includes two concentric circles. The inner circle represents wheat chromosome 3AS (green) and orthologous chromosomes from rice (blue), *Brachypodium* (yellow) and *Sorghum* (red). The outer circle illustrates the heat map and colored bars indicate the density of BLAST hits per mega base of sequence (bin size = 1 Mb). Colored lines in the center represent the putative orthologous relationship between the wheat chromosome 3AS and rice chromosome 1, *Brachypodium* Bd2 and *Sorghum* chromosome 3.

Repeat-masked BESs were used for a comparative analysis with other model grass genomes. The 3AS BESs were subjected to a similarity search against rice chromosome 1, *Brachypodium* chromosome 2 and *Sorghum* chromosome 3 and also to chromosome 3B BESs using BLASTN. There were 193 hits to rice chromosome 1, 346 on *Brachypodium* chromosome 2 and 185 to *Sorghum* chromosome 3 (Figure 3, Additional file 6). There were 632 hits to 3B BESs demonstrating a high level of homology, as expected. The 3A BESs, when aligned to a model grass genome based on sequence similarity and ordered based on *Brachypodium*, showed a mosaic of synteny (Figure 3). The short arm of chromosome 3A showed a high level of synteny with short arm of rice chromosome 1, *Sorghum* chromosome 3 and *Brachypodium* chromosome 2 (Figure 3). Sequence hits were distributed all along chromosome 3AS with a mean of 0.9 hits/Mb of sequence and range of 0–27 hits/Mb of sequence, however, the density of hits were higher towards the distal end of the chromosome, which could be due to higher gene density in terminal part of the chromosome [24,43]. A similar trend of increased gene density towards the distal end of the chromosome was seen in chromosome 3B [24]. The BESs hit 43 bins (bin size = 1 Mb) in rice chromosome 1 with a mean of 4.4 hits per bin and range of 0–21 hits per Mb of sequence. In *Brachypodium* chromosome 2, the mean number of hits per Mb of sequence was 6.9 with range of 0–31 hits/Mb of sequence. In *Sorghum* chromosome 3, the mean hits per Mb of sequence were 2.4. Our comparative analysis validates the high synteny (70-77%) between the short arm of chromosome 3A with the short arms of rice chromosome 1, *Brachypodium* chromosome 2 and *Sorghum* chromosome 3 (Figure 3).

### **Molecular markers from 3AS**

In order to identify new putative markers for physical and genetic mapping, 17,591 BESs were analyzed for the presence of SSRs, which could be converted into microsatellite markers. A total of 1,057 perfect repeats of di-, tri- and tetranucleotide repeats were identified. The frequency of SSRs derived from the BESs was about one SSR per 10.4 kb (Table 3). The most abundant of these SSRs were dinucleotide 779 (73.8%) and trinucleotide 268 (25.2%); tetranucleotides were rare (1.0%). Nearly 28% of SSRs (both di- and trinucleotide) were AT rich (i.e., TA, AT and TAA). A comparison of the frequency and distribution of the SSRs between the A and B genomes of wheat indicates a higher density of SSRs on chromosome 3B (one SSR per 6.1 kb) compared to chromosome 3AS (one SSR per 10.4 kb). Furthermore, the dinucleotide motif (TA)_n_ was most abundant (12.6%) on chromosome 3AS in contrast to the trinucleotide motif (AAG), which was the most abundant in wheat chromosome 3B [9]. Other species, such as *Brachypodium*, rice and maize, also have a higher frequency of trinucleotide motifs, however, species such as papaya show a higher frequency of dinucleotide motifs and soybean had higher frequency of tetranucleotide motifs [44]. In our study, the frequency of SSRs on chromosome 3AS is far lower than that of chromosome 3B. The higher frequency of AT-rich di- and trinucleotide motifs in wheat and maize suggests that AT-rich region are associated with repetitive sequences, in contrast to GC-rich regions, which are associated with the transcribed part of the genome.

Microsatellite primer pairs were successfully developed from 758 out of 1,057 SSRs. Of the 758 SSRs primer pairs, 155 were developed from class I microsatellites with more than nine motif repeats (Additional file 7). The BESs were also processed to identify junctions between TE and other sequences using ISBPfinder.pl [45]. In total, 7,928 insertion sites were identified in 17,591 BESs, and primer pairs were successfully designed from 695 suitable junctions (Additional file 7). A repeat was identified every 1.39 kb on t3AS, whereas a repeat was identified every 0.89 kb on t1AL [13], suggesting that repeat-based makers could play an important role in the saturation mapping of the wheat genome. A set of 96 class I SSRs and 96 ISBP primer pairs were tested by PCR amplification for 3AS specificity and utility across species.

**Figure 4:**
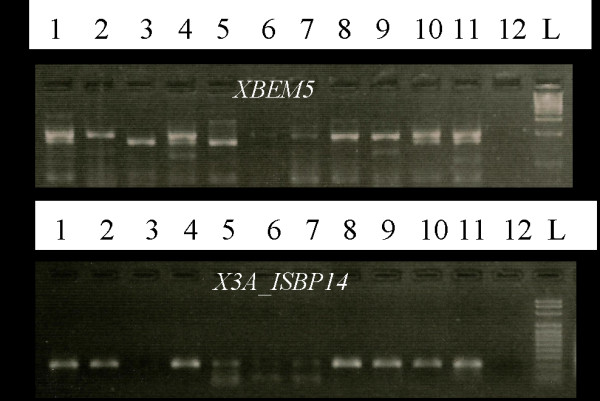
** Example of the localization SSR and ISBP markers on chromosome 3AS and amplification across species.** PCR products separated on 3 % agarose gels after amplification with one SSR (*Xbem5*) and one ISBP (*Xisbp3A-14*). Templates were DNA from (1) CS wheat, (2) a 3AS BAC library superpool, (3) N3AT3B, (4) Dt3AS, (5) Dt3AL, (6) *T. monococcum* subsp. *monococcum* TA4342-L96, (7) *T. monococcum* subsp. *aegilopoides* TA4342-L95, (8) *T. turdigum* subsp*. durum* cv. Langdon, (9) Langdon DIC-3A, (10) *T. aestivum* cv. Opata 85, (11) synthetic wheat W79849, (12) water, (L) 100-bp DNA ladder.

For testing 3AS specificity, primer pairs were amplified on genomic DNA of a 3AS BAC pool, Chinese Spring (CS), and the CS aneuploids N3AT3B (nullisomic for 3A and tetrasomic for 3B), Dt3AS (nullisomic 3AL) and Dt3AL (nullisomic 3AS). An ISBP primer pair was considered 3AS specific if it did not amplify a product from N3AT3B or Dt3AL but amplified products from all other stocks (Figure 4). Nearly 18% of the SSRs did not produce amplicons from N3AT3B or Dt3AL suggesting these markers had 3AS specificity (Figure 4). Twenty-nine percent of the ISBP primer pairs did not amplify from N3ATB or Dt3AL indicating their specificity to 3AS (Figure 4). However, all the ISBP and ISSR markers that amplified from CS (93% SSRs and 98% ISBPs) also amplified from the CS t3AS BAC library super pools, suggesting that they are present on chromosome 3AS. The nonspecificity of nearly two-thirds of the ISBP and four-fifths of the SSRs markers may be attributed to a combination of several factors including amplification from the homoeologous chromosomes, t3AS has a portion of the long arm of chromosome 3A, flow-sorted chromosome arm-based libraries have a reported 10-13 % contamination [3]; duplications on other wheat chromosomes, because microsatellites have a high possibility of being moved by transposable elements; or bias in the sample tested.

Nearly, 53% (23/44) of ISBPs designed from 1AL BESs were 1AL specific [13], suggesting that because the first primer is designed from repeat and the second one from unique sequences, the high specificity of the unique sequences becomes very significant for specific amplification.

The set of 96 class I SSRs and 96 ISBP primer pairs were also tested by PCR amplification on wheat genotypes, the parents of three mapping populations: cultivated diploid wheat (*T. momococcum* subsp. *monococcum*) and wild diploid wheat (*T. monococcum* subsp. *aegilopoides*); tetraploid wheat (*T. turgidum* subsp. *durum*) genotypes Langdon and Langdon DIC-3A; and bread wheat genotypes CS, Opata 85 and synthetic W7984 (Figure 4). More than 93% of the SSR and 97% of ISBP primer pairs gave amplification from diploid, tetraploid and hexaploid wheat. Twenty-six percent (25/96) ISBPs and 16% (15/96) SSRs were found polymorphic between parents of diploid mapping population *T. monococcum* and *T. aegilopoides* on 3% agarose gels. To eliminate the effect of amplification from 3B and 3D, we are mapping these markers in a diploid wheat (AA) *T. monococcum* subsp. *monococcum/*subsp. *aegilopoides* RIL population (data not shown). Amplification of the primer pairs from diploid, tetraploid and hexaploid wheat shows their reproducibility and wide adaptability of these markers.

In summary, a total of 1,453 new (758 SSR + 695 IBSP) putative markers have been developed spanning the 3AS arm, which adds over 200% more markers than mapped in all previous studies (<600 markers) on this arm. The universality and variability of SSRs make these sequences an attractive source of developing microsatellite markers. However, because most of the genome (>80%) is repetitive, the ISBP repeat insertion-based markers could be very important in saturating markers in this portion of the genome and, thus, could enhance the efficiency of breeding programs. The potential of such repeat-based markers in crop breeding for high-throughput, marker-assisted selection has been suggested by Paux et al. [45]. More than 30 genes and QTLs related to domestication traits, such as brittle rachis; tillering; disease resistance genes against stem rust, leaf rust, tanspot, and *Septoria tritici* blotch; and agronomic traits such as preharvest sprouting and root-shoot biomass, have been genetically mapped on chromosome 3AS of wheat (http://www.shigen.nig.ac.jp/wheat/komugi/genes/symbolClassList.jsp) [46]. In addition to the 1,453 molecular markers (SSR and ISBP) developed in the present study, we have obtained sequence tags of 161 of the estimated ~2,850 genes on chromosome 3AS. The 1,453 molecular markers plus 161 sequence tags of genes will aid in marker-assisted selection and the map-based cloning of above-mentioned agronomic traits on 3AS arm. The molecular markers will also help to align the physical and genetic maps of chromosome 3AS.

## **Conclusion**

BESs are a valuable genomic resource for genome organization analysis, comparative genomics and marker development. Analysis of 17,591 3AS BESs revealed differences in the repeat landscape of the A- and B-genome homoeologous chromosomes and estimate of gene content, *Triticum*-specific and genome-specific genes. We also developed BESs-derived microsatellite and ISBP putative markers as a resource for integrating physical and genetic maps and for mapping and cloning of a large number of agronomically important genes on 3AS arm.

## **Methods**

### **BAC library**

We used the first of the two BAC libraries constructed from the short arm of chromosome 3A (3AS). The library (code TaaCsp3AShA) consists of 55,296 clones arranged in 144 384-well plates. With the average insert size of 80 kb and 11% contamination with other chromosomes, the library represents 10.9x equivalents of the chromosome arm [25].

### **BAC end sequencing**

Twenty-six 384-well plates (9,984 BAC clones) randomly selected from the 3AS library were end sequenced (5’ & 3’) at the J. Craig Venter Institute (Rockville, MD). BAC clones of *Hin*dIII 96-deep-well blocks containing 1.2 ml/well of 2xYT medium were grown overnight at 37°C with shaking at 300 rpm. The cells were harvested by centrifugation, and the BAC DNA was purified using a REAL Prep 96 Plasmid Kit (Qiagen, Valencia, CA). For BAC-end sequencing, 5 μl of purified BAC DNA (~ 0.2 to 0.5 μg) was used in a sequencing reaction with ABI BigDye terminators (Applied Biosystems, Foster City, CA). Template DNA was sequenced from both directions with pCC1BAC/pIndigoBAC-5 Forward and Reverse End-Sequencing Primers (Epicentre, Madison, WI). Electrophoresis of the sequencing reaction was carried out with a 3730xl DNA Analyzer (Applied Biosystems, Foster City, CA).

### **Sequence data processing**

The sequences and quality files from trace files were read by the Phred program for base calling and trimmed to remove vector and low quality bases [28,29]. The high-quality sequence data were then filtered for sequences contaminated with *Escherichia coli* or with plant organelle genomes based on matches to the wheat mitochondria and chloroplast sequences.

### **Sequence analysis**

The high-quality BESs were compared with several repeat databases, including the Triticeae repetitive (TREP) sequence database (http://wheat.pw.usda.gov/ITMI/Repeats/)[47, the TIGR plant repeat database (ftp://ftp.tigr.org/pub/data/TIGR_Plant_Repeats/), and RepBase at GIRI. (http://www.girinst.org/)[48]. Self-BLASTN and BLASTX at an *e-*value cutoff of 10^−5^ was used [33]. The composition and contents of repeat element in BESs was assessed using the RepeatMasker program (http://www.repeatmasker.org/) with the default settings using each of the above databases as the custom library option. Finally, a cutoff score of 250 was used to identify the repetitive sequences. The BESs were annotated based on their best match to the repeat database and categorized according to (http://wheat.pw.usda.gov/ITMI/Repeats/gene_annotation.pdf)[47]. Sequences matching known repeats were masked as N and repeat masked sequences were further used to identify novel repeats. Self-BLASTN was performed on repeat-masked BESs to identify sequences that had multiple strong matches to other BESs with an *e*-value <10^−50^. The blast output file was processed using RECON software (http://selab.janelia.org/recon.html) [49] to identify families with eight or more sequences. The sequences were extracted and aligned by ClustalW [50]. Consensus sequences for each family with a minimum 100-bp alignment and 80 % identity were selected. The consensus sequences were compared to each other and aligned using the CAP3 software [32] to merge overlapping regions and extend the sequences. The putative novel repeats were searched in BLAST against nonredundant nucleotide, EST and protein databases to remove any characterized sequences. The repeat-masked sequences were subjected to gene content analysis by a homology search using TBLASTX versus the *T. aestivum* Transcript assembly 2.0 (e-value 10^−50^) (http://www.jcvi.org/wheat/downloads.php). The cumulative match lengths were used to calculate the coding fraction, as described for repetitive elements. The repeat masked sequences were subjected to Blast2GO for functional annotation using default parameters [37].

### **Comparative sequence analysis**

The rice (http://rice.plantbiology.msu.edu/), *Brachypodium* (http://ftp.brachypodium.org/) and *Sorghum* (http://www.phytozome.net/sorghum) sequences were downloaded from their respective databases. The 3B BESs were obtained from NCBI Genbank and 3B unigene sequences were requested from authors [24]. The repeat-masked BESs sequences were searched in model grass genomes by BLASTN against *Brcahypodium* chromosome 2, rice chromosome 1 and *Sorghum* chromosome 3 at an e-value of 1e-5 and a minimum hit length of 50 bp. The unique hits for each BESs were used for developing a circos map for visualization of synteny [51]. The 3AS BESs were compared with chromosome 3B unigenes, barley full length cDNA by BLASTN at an e-value of 1e-10.

### **Development of molecular markers from BESs**

The BESs were screened computationally for the presence of di-, tri- and tetranucleotides SSRs using the SSRIT program (ftp://ftp.gramene.org/pub/gramene/software/scripts/ssr.pl). SSRs motifs that span ten or more nucleotides were recorded, and SSR location data in sequence were extracted from the SSR search output files. Sequences containing SSR were used to design primers using an in-house perl script and Pimer 3.0 with default settings, and ISBP primers were designed using IsbpFinder.pl [45]. All the SSR and ISBP primer pairs were amplified using the 0.8 μM of primer and 0.25 mM of dNTP, 1.25 mM MgCl_2_, 0.1 U Taq polymerase (Bioline, CA, USA) with 1× buffer and 50 ng template DNA. PCR amplification was conducted using the following procedure: nine cycles (95°C for 30 sec, 58°C for 60 sec minus 0.5°C each cycle, 72°C for 60 sec), 25 cycles (95°C for 30 sec, 53°C for 45 sec, 72°C for 60 sec) and one additional cycle (72°C for 7 minutes). Genomic DNA amplified with SSR and ISBP primers were separated on 3 % GenePure HiRes Agarose (ISC Bioexpress, Kaysville, UT, USA) gels.

## **Abbreviations**

BAC, bacterial artificial chromosome; BESs, BAC end sequences; EST, expressed sequence tag; GO, gene ontology; HSP, high-scoring segment pair; IB, isolation buffer; Gb, Gigabase; kb, kilobase; Mb, Megabase; bp, base pair; MDA, multiple displacement amplification; SSR, simple sequence repeat; ISBP, insertion site based polymorphism; RIL, recombinant inbred line..

## **Author’s contributions**

BSG and WL: conceived the study; SKS and WL: carried out the sequence data analysis; PR and AC: BAC-end sequencing; HS and JD: provided the BAC library and participated in manuscript preparation; SKS, BSG and WL wrote the manuscript. All authors read and approved the final manuscript.

## Supplementary Material

**Additional file 1** Sequence for putative novel repeats identified from 3AS BAC end sequences. Click here for file

**Additional file 2** ** Novel repeats, length and number of hits identifiedin 3AS BAC end sequences.** Click here for file

**Additional file 3** Blast2GO annotations of repeat masked BESs. Click here for file

**Additional file 4** Class wise distribution of repetitive elements in homoeologous genomes of hexaploid wheat based on BESs analysis. Click here for file

**Additional file 5** Comparative analyses of wheat chromosomes 3AS BESs and 3B unigenes. Click here for file

**Additional file 6** ** Microsynteny between wheat chromosome 3A and rice chromosome 1,*Brachypodium*chromosome 2 and*Sorghum*chromosome 3.** Click here for file

**Additional file 7** SSR and ISBP markers developed from BESs. Click here for file
